# Dietary β-glucan supplementation mitigates heat stress-induced impairment of performance, egg quality, and intestinal integrity in laying hens

**DOI:** 10.14202/vetworld.2026.2634-2649

**Published:** 2026-06-28

**Authors:** Mohannad Abuajamieh, Zeinab M. H. Mahasneh, Mohmmad Al-Qaisi, Mohamed A. Abedal-Majed, Abdur-Rahman Al-Fataftah, Anas Abdelqader

**Affiliations:** 1 Department of Animal Production, School of Agriculture, The University of Jordan, Amman 11942, Jordan; 2 Department of Animal Production and Protection, Faculty of Agriculture, Jerash University, Jerash 26150, Jordan

**Keywords:** beta-glucan, egg quality, gut integrity, heat stress, intestinal morphology, laying hens, performance, *Saccharomyces cerevisiae*

## Abstract

**Background and Aim:**

Heat stress (HS) severely impairs laying hen productivity, egg quality, and intestinal integrity, posing significant challenges to poultry production under changing climate conditions. This study evaluated the dose-dependent effects of dietary yeast-derived β-glucan (BG) supplementation as a nutritional strategy to mitigate HS-induced impairments in commercial Hy-Line laying hens.

**Materials and Methods:**

One hundred and sixty 22-week-old Hy-Line W-80 laying hens were randomly assigned to four dietary treatments (0, 1, 2, or 3 g BG/kg feed; n=40 hens/treatment) for a 28-day pre-feeding adaptation period. Hens were then subjected to two experimental periods: Period 1 (7 days under thermo-neutral conditions, 25.7 ± 2.0°C) and Period 2 (4 days of cyclic HS, 38.4 ± 0.8°C for 4 h/day). Performance parameters (feed intake, egg production, egg mass, feed conversion ratio [FCR]), egg quality traits, blood physiological and biochemical indicators, and intestinal morphometry (jejunum and ileum) were assessed.

**Results:**

Cyclic HS significantly reduced feed intake, laying rate, egg mass, and impaired egg quality (albumen height, Haugh unit, shell thickness, yolk index) while increasing FCR and causing intestinal villus atrophy (p < 0.01). Supplementation with 3 g/kg BG during HS improved laying rate (64% vs. 55.7%), egg mass (36.5 vs. 29.1 g/hen/day), and FCR (2.3 vs. 2.8) compared to unsupplemented controls (p < 0.01). A treatment × period interaction was observed for yolk index (p = 0.05), with BG3 showing superior values under HS. Notably, 3 g/kg BG increased jejunal and ileal villus height under HS (by 10% and 7% vs. control, respectively; p < 0.01), thereby preserving gut integrity. No adverse effects on blood gases or biochemical parameters were noted.

**Conclusion:**

Dietary supplementation with 3 g/kg yeast-derived β-glucan (25% 1,3-β-glucan) effectively mitigated short-term cyclic HS effects by enhancing productive performance, feed efficiency, yolk index, and intestinal morphology in laying hens. These findings highlight higher-dose BG as a practical strategy to support gut health and productivity during heat waves.

## INTRODUCTION

Global food security is a major concern for humanity, as the primary sources of nourishment (plants and animals) are influenced by numerous factors, including diseases, management practices, genetic advancements, and environmental conditions. Future global population growth is expected to be accompanied by an estimated 70% increase in food demand [1]. Failure to align food production with this increasing demand may pose serious challenges to global food security. To sustain the growing population, it is essential to develop and implement innovative strategies that enhance both plant and animal productivity while reducing food losses and waste.

Several factors influence livestock health and production, among which climate change, particularly heat stress (HS), has emerged as a major challenge. Climate change is increasingly recognized as a significant constraint to livestock production because it adversely affects both the quality and quantity of animal-derived products. Even in production systems with well-established infrastructure that meets international standards, the effects of global warming and HS continue to limit the full genetic potential of farm animals. HS poses a substantial threat to animal productivity, welfare, and health. During summer, ambient temperatures often exceed the thermal tolerance of livestock and poultry, leading to reduced feed intake, decreased egg production, impaired egg quality, and increased feed conversion ratio (FCR). Previous studies have demonstrated that short-term HS can significantly reduce feed intake by approximately 28 g/hen and egg production by approximately 30% under acute conditions [2]. More severe and prolonged HS further exacerbates these effects, resulting in reductions in egg production exceeding 50% during extended exposure periods [3]. Consequently, HS contributes to substantial economic losses during heat waves [4–6]. These reductions in animal productivity can markedly affect farm profitability, particularly for small-scale producers. Therefore, there is a continuous need to identify and implement effective nutritional and management strategies to mitigate the detrimental effects of HS.

In recent decades, the negative effects of HS on farm animals have become increasingly pronounced. Climate projections suggest that global temperatures will continue to rise in the coming decades, potentially increasing the frequency, duration, and severity of HS events [5, 7]. In laying hens, cyclic HS has been reported to reduce egg production, decrease intestinal villus height, and alter physiological responses, collectively indicating impaired nutrient utilization and reduced productive performance [8]. Consequently, the development of nutritional strategies to improve resilience to thermal stress has become an important research priority.

One promising nutritional intervention is dietary supplementation with β-glucan (BG), a non-starch polysaccharide naturally present in cereals, yeast, fungi, and bacteria [9, 10]. β-glucan possesses prebiotic and immunomodulatory properties and has been shown to suppress pathogenic bacteria such as *Escherichia coli* while supporting gut barrier integrity [11–13]. Previous studies have also reported that BG supplementation can reduce circulating lipopolysaccharide (LPS) concentrations, an endotoxin associated with intestinal barrier dysfunction and systemic inflammation [10, 14]. Furthermore, BG has been shown to promote the proliferation of beneficial intestinal microorganisms and improve gut microbial balance in various animal species [12, 15]. In addition, BG has demonstrated potential for modulating inflammatory responses in broiler chickens challenged with coccidiosis [16].

Although BG supplementation has shown beneficial effects in broilers exposed to HS and in laying hens receiving relatively low dietary concentrations (100–200 mg/kg) during rearing combined with early heat-conditioning programs [17], important knowledge gaps remain. Specifically, the effectiveness of higher dietary inclusion levels (1–3 g/kg) administered directly during the laying phase under cyclic HS conditions has not been thoroughly investigated. Moreover, most studies evaluating nutritional mitigation strategies for HS in laying hens have focused primarily on antioxidants, electrolytes, or vitamins, whereas information regarding BG supplementation during active egg production remains limited. In addition, few studies have simultaneously examined productive performance, egg quality, physiological responses, and intestinal morphometric characteristics under controlled cyclic HS conditions. Therefore, whether higher dietary concentrations of BG can preserve intestinal integrity and sustain productive performance during acute thermal challenges remains unclear.

Based on previous evidence, we hypothesized that dietary BG supplementation could improve productive performance, physiological responses, and intestinal morphology in laying hens exposed to cyclic HS by supporting gut barrier integrity and reducing endotoxin translocation. The present study was designed to provide a comprehensive evaluation of the role of BG supplementation during the laying phase, a period characterized by high metabolic demand and susceptibility to environmental stressors.

Unlike previous investigations that primarily evaluated lower BG inclusion levels or focused on earlier production stages, this study assessed higher dietary BG concentrations (1–3 g/kg) administered directly during the laying phase under controlled cyclic HS conditions. Furthermore, the study integrated measurements of productive performance, egg quality assessment, blood physiological and biochemical analyses, and quantitative intestinal morphometry to provide a comprehensive understanding of the mechanisms by which BG may alleviate HS-induced impairments.

Therefore, the specific objective of this study was to determine the dose-dependent effects of dietary BG supplementation on productive performance, egg quality, blood physiological and biochemical parameters, and intestinal morphology under thermo-neutral (TN) and cyclic HS conditions. We further sought to evaluate whether higher dietary BG levels could preserve gut structural integrity and support productivity in commercially relevant laying hens exposed to acute thermal stress. The findings of this study are expected to contribute to the development of practical nutritional strategies to improve gut health, resilience, and productive efficiency in laying hens under both TN and HS conditions.

## MATERIALS AND METHODS

### Ethical approval

All experimental procedures involving animals were conducted in accordance with the principles of animal welfare and the internationally accepted guidelines for the care and use of animals in research. The study protocol, including animal housing, management, handling, HS induction, blood sampling, and euthanasia procedures, was reviewed and approved by the Animal Ethics Committee of the Scientific Research Support Fund, Higher Council for Science and Technology (HCST), Amman, Jordan (Approval No. AGR/1/05/2021).

Throughout the experimental period, the health and welfare status of the laying hens were monitored daily by trained personnel. Environmental conditions, stocking density, feeding, and watering systems were maintained in accordance with standard poultry husbandry practices to minimize unnecessary stress. The HS protocol was designed to simulate commercially relevant environmental conditions while avoiding excessive animal suffering. Birds were observed continuously during HS exposure for signs of severe distress, and appropriate intervention measures were available if required. No mortality or welfare-related complications occurred during the study.

Blood collection was performed by trained personnel using standard veterinary procedures to minimize handling time and discomfort. At the conclusion of the experiment, birds selected for tissue collection were humanely euthanized by cervical dislocation followed by exsanguination, in accordance with approved ethical guidelines. All efforts were made to minimize animal suffering and to use the minimum number of animals necessary to achieve scientifically valid results.

### Study period and location

The experiment was conducted from August to October 2024 at the Environmental Physiology Laboratory, School of Agriculture, The University of Jordan, Amman, Jordan. The study consisted of a 28-day dietary adaptation period followed by two experimental environmental phases: a 7-day TN period and a 4-day cyclic HS challenge.

### Study design

A total of 160 Hy-Line W-80 laying hens aged 22 weeks, with an average body weight of 1,257 ± 15 g, were obtained from a commercial poultry farm and transferred to the Environmental Physiology Laboratory, The University of Jordan. Individual body weights were recorded before allocation to ensure comparable initial body weights among treatments.

The hens were randomly assigned to four dietary treatments and housed in four pens, each containing eight cages (0.75 × 0.75 × 0.35 m), providing a stocking density of 1125 cm²/hen. Each treatment consisted of eight replicates with five hens per replicate.

The dietary treatments were as follows:

1. Basal diet (Ctrl)
2. Basal diet supplemented with 1 g/kg BG (BG1)
3. Basal diet supplemented with 2 g/kg BG (BG2)
4. Basal diet supplemented with 3 g/kg BG (BG3)

To minimize experimental bias, all samples and recorded data were coded before analysis. Personnel responsible for data collection and measurements were blinded to treatment allocation throughout the study.

A 28-day pre-feeding period was implemented before the environmental challenge phases to allow sufficient time for BG supplementation to exert potential effects on intestinal morphology, physiological responses, and productive performance. The diets used during the adaptation phase were identical to those used during the experimental periods.

Following the adaptation period, hens were exposed to two environmental phases. Period 1 (P1) consisted of 7 days under TN conditions (25.7 ± 2.0°C). Period 2 (P2) consisted of 4 consecutive days of cyclic HS (38.4 ± 0.8°C for 4 h/day).

The cyclic HS protocol was designed to simulate acute heat-wave conditions commonly encountered in commercial poultry production systems. This experimental design is distinct from previous studies because cyclic HS was applied directly during the laying phase following dietary adaptation to varying BG inclusion levels (up to 3 g/kg), whereas earlier studies primarily focused on early-life heat acclimation or chronic HS exposure.

The BG product was derived from *Saccharomyces cerevisiae* cell wall extracts (ICC Brazil Co., Ltd., São Paulo, Brazil) and contained 25% 1,3-BG according to the manufacturer’s specifications. The product was incorporated into experimental diets to produce the designated BG treatments. The BG concentration of the finished diets was not analytically verified and was based on formulated inclusion levels provided by the manufacturer.

Hens were maintained under a controlled lighting schedule of 16 h light and 8 h dark. Feed was supplied through linear feeders providing approximately 10 cm feeder space per hen, whereas water was supplied through nipple drinkers (three nipples per five hens).

All birds had been beak-trimmed at the source farm before initiation of the study. Experimental diets were formulated and manufactured at a commercial feed mill for the entire study period. Diet composition and nutrient analysis are presented in Table 1. Different-colored bags were used to store the diets to minimize mixing errors and facilitate identification.

**Table 1:** Ingredients and analyzed diet composition of the laying hens.

Ingredient	Unit	Layer feed
Ground corn	%	59.11
Soybean meal (48% crude protein)	%	21.80
Calcium carbonate	%	10.00
Wheat bran	%	6.00
Vegetable oil	%	1.20
Sodium chloride	%	0.20
Monocalcium phosphate	%	0.60
DL-methionine	%	0.35
L-lysine HCl	%	0.20
Sodium bicarbonate	%	0.20
Mycotoxin binder	%	0.05
Vitamin and mineral premix	%	0.20
Phytase	%	0.09

**Diet analysis**		

**Parameter**	**Unit**	**Value**

Total	kg	1000.00
Dry matter	%	88.60
Crude protein	%	17.20
Metabolizable energy	kcal/kg	2850.00
Calcium	%	3.65
Phosphorus	%	0.45
Digestible lysine	%	0.80
Digestible methionine + cysteine	%	0.75
Digestible threonine	%	0.45
Digestible valine	%	0.73
Digestible tryptophan	%	0.13
Sodium	%	0.13
Chloride	%	0.26

During the HS phase, rectal temperature was measured twice daily in a subset of hens (n = 2 hens/replicate; 16 hens/treatment). Measurements were obtained using a standard digital thermometer, which was allowed to stabilize for 1 min before recording.

No mortality or culling occurred during the experiment. Birds were monitored daily, and no welfare concerns other than expected panting responses during HS exposure were observed.

The vitamin premix provided the following per kilogram of diet: 200,000 IU vitamin A (retinol acetate), 50,000 IU vitamin D3, 500 mg vitamin E (α-tocopherol acetate), 60 mg vitamin K3, 40 mg vitamin B1, 100 mg vitamin B2, 240 mg D-pantothenate, 100 mg vitamin B6, 0.3 mg vitamin B12, 800 mg niacin, 1 mg biotin, 1,200 mg folic acid, 1,200 mg iron, 200 mg cobalt, 1,600 mg zinc, 1,600 mg manganese, 20 mg iodine, 4 mg selenium, and 10,000 mg choline chloride.

### Data collection

Experimental data were collected during two chronological phases: the TN phase (P1; 7 days) and the HS challenge phase (P2; 4 days). During P1, all hens were maintained under TN conditions (25.7 ± 2.0°C; relative humidity 48 ± 5%).

**Figure 1:**
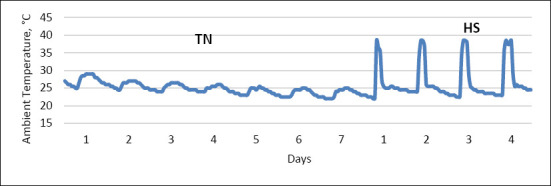
Ambient temperature profile during thermo-neutral (TN) and cyclic heat stress (HS) conditions.

Following P1, all treatment groups were exposed to cyclic HS for 4 consecutive days. The HS regimen consisted of 24.2 ± 1.8°C and 48.1% relative humidity between 03:00 PM and 11:00 AM, and 38.4 ± 0.8°C and 33.6% relative humidity between 11:00 AM and 03:00 PM (Figure 1). Ambient temperature was recorded hourly using a data logger. Relative humidity and air velocity within the chambers were not experimentally controlled and therefore reflected ambient environmental conditions.

### Productive performance

Eggs were collected daily from all treatment groups. Individual egg weight, egg mass, and hen-day laying rate were determined.

Hen-day egg production (%) was calculated as:

Number of eggs produced per day/number of hens × 100.

Egg mass (g/hen/day) was calculated as average egg weight × hen-day egg production percentage.

Daily feed intake and FCR were calculated for each treatment throughout the study.

For egg quality assessment, 30 eggs per replicate were randomly collected during each experimental period. Albumen height, yolk color, Haugh unit, breaking strength, eggshell thickness, yolk height, yolk diameter, and yolk index were determined using a digital egg tester (DET 6500, NABEL Co., Ltd., Kyoto, Japan).

### Blood parameters

Blood samples were collected at the end of each experimental period, immediately after HS exposure during P2. Hens were fasted for 3 h before sampling.

Four blood samples were collected from each replicate (32 samples/treatment) through jugular venipuncture into ethylenediaminetetraacetic acid-treated tubes (Vacuette tube, Greiner Bio-One, Austria). Samples were collected in a balanced alternating sequence across treatments. Blood samples were centrifuged at 1,200 × *g* for 15 min at 4°C and stored at −20°C until analysis.

Whole blood gas analysis was performed immediately after collection using an epoc® Blood Analysis System (Siemens Healthineers GmbH, Erlangen, Germany). Measurements included pH, partial pressure of carbon dioxide (pCO_2_), partial pressure of oxygen (pO_2_), bicarbonate (HCO_3_), total CO_2_, potassium, chloride, sodium, ionized calcium, hemoglobin, hematocrit, and oxygen saturation. All measurements were conducted at ambient temperature without additional temperature correction.

### Blood biochemical parameters

Thirty-two samples per treatment were analyzed for haptoglobin, alkaline phosphatase, LPS, insulin, serum amyloid A, alanine transaminase, and glutathione peroxidase using enzyme-linked immunosorbent assay kits (ELK Biotechnology Co., Wuhan, China) according to the manufacturer’s instructions.

Blood glucose, lactate, and blood urea nitrogen concentrations were determined using enzyme-linked immunosorbent assay spectrophotometric kits (MTD Diagnostics, S. Nicola La Strada, CE, Italy) according to the manufacturer’s instructions.

Assay sensitivity and intra-assay/inter-assay coefficients of variation were within the ranges specified by the manufacturers.

### Post-mortem tissue collection

At the end of the experiment, hens were humanely euthanized by cervical dislocation followed by jugular exsanguination. Intestinal samples were collected from standardized anatomical locations. Jejunal samples were obtained 10 cm distal to the end of the duodenal loop, whereas ileal samples were collected 10 cm proximal to the ileocecal junction. Segments measuring approximately 2–3 cm were flushed with cold phosphate-buffered saline to remove luminal contents and fixed in 10% neutral-buffered formalin for histological evaluation.

### Intestinal histology

One hen per replicate was sampled for intestinal histological evaluation. Jejunal and ileal samples were fixed in formalin and processed at the Histology Laboratory, School of Medicine, The University of Jordan. Tissues were sectioned and stained with hematoxylin and eosin stain.

For each intestinal segment, three slides per hen were prepared, and five non-overlapping, well-oriented microscopic images were captured per slide at 100× magnification. At least five intact villi and associated crypts were measured from each image. Measurements were averaged to generate a single representative value per hen. Damaged, folded, or obliquely sectioned villi were excluded from analysis.

Image acquisition and morphometric analysis were performed using Leica LAS EZ 3.4 software (Leica Microsystems, Wetzlar, Germany). To minimize observer bias, all morphometric evaluations were conducted in a blinded manner.

### Statistical analysis

All data were analyzed using the MIXED procedure of SAS software version 9.4 (SAS Institute Inc., Cary, NC, USA). The statistical model included dietary treatment, environmental period (TN versus HS), and their interaction as fixed effects, whereas pen was considered the experimental unit and included as a random effect. Means were compared using Tukey’s adjustment for multiple comparisons. Sample size selection was based on previous studies. Statistical significance was declared at p ≤ 0.05, whereas values of 0.05 < p ≤ 0.10 were considered trends.

## RESULTS

### Rectal temperature

**Figure 2:**
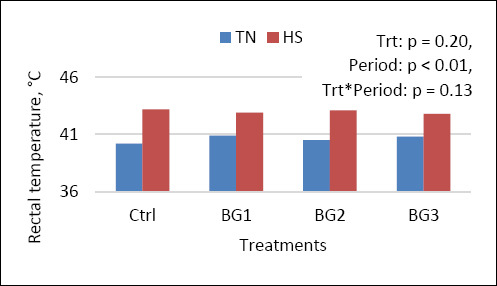
Effects of β-glucan (BG) supplementation on rectal temperature of laying hens maintained under thermo-neutral (TN) or cyclic heat stress (HS) conditions. Ctrl = control hens receiving the basal diet, BG1 = hens receiving 1 g/kg BG, BG2 = hens receiving 2 g/kg BG, and BG3 = hens receiving 3 g/kg BG. Data are presented as least square means (LSM) ± standard error of the mean (SEM). No significant differences were observed among BG treatments under HS conditions (p > 0.05).

A significant main effect of period was observed for rectal temperature, with hens exposed to HS exhibiting higher rectal temperatures than those maintained under TN conditions (43.0 vs. 40.6°C; p < 0.01; Figure 2), indicating that the HS challenge was successfully established. No significant treatment × period interaction was detected (p = 0.13), indicating that BG supplementation did not significantly influence rectal temperature under either environmental condition. However, a numerical reduction of approximately 0.3°C was observed in BG-supplemented hens under HS conditions compared with the control treatment (Figure 2).

### Productive performance

**Figure 3:**
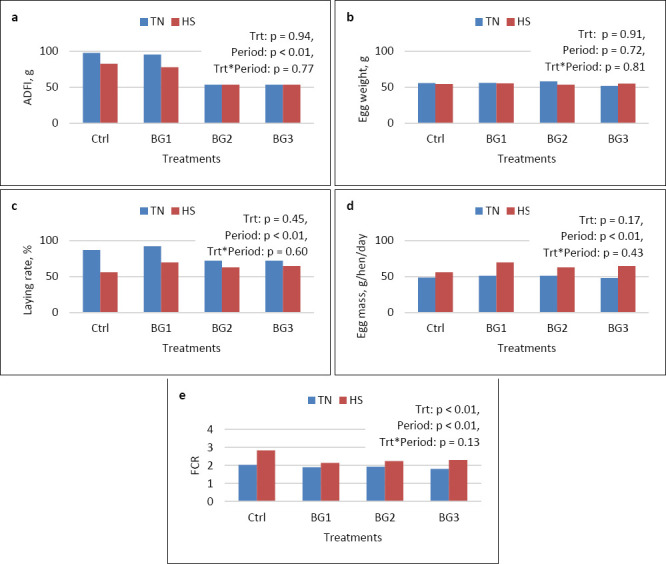
Effects of β-glucan (BG) supplementation on (a) feed intake, (b) egg weight, (c) laying rate, (d) egg mass, and (e) feed conversion ratio (FCR) of laying hens maintained under thermo-neutral (TN) or cyclic heat stress (HS) conditions. Ctrl = control hens receiving the basal diet, BG1 = hens receiving 1 g/kg BG, BG2 = hens receiving 2 g/kg BG, and BG3 = hens receiving 3 g/kg BG. Data are presented as LSM ± SEM.

The effects of environmental conditions and BG supplementation on productive performance in laying hens are presented in Figure 3. As expected, HS markedly reduced feed intake, laying rate, and egg mass (81.6 vs. 95.6 g/hen/day, 63% vs. 86%, and 34.45 vs. 49.47 g/hen/day, respectively; p < 0.01; Figures 3a, c, and d) and increased FCR (2.38 vs. 1.91; p < 0.01; Figure 3e) compared with TN conditions. Notably, BG-supplemented hens exhibited significant improvements in laying rate, egg mass, and FCR under HS conditions (p < 0.05), demonstrating a dose-dependent response pattern that has been less commonly reported in laying hen studies evaluating BG concentrations below 0.5 g/kg.

### Egg quality

HS adversely affected both external and internal egg quality characteristics. Albumen height, Haugh unit, eggshell thickness, yolk height, and yolk index were significantly reduced under HS conditions compared with TN conditions (5.6 vs. 8.6 mm, 75.4 vs. 93.3, 0.34 vs. 0.36 mm, 15.8 vs. 17.2 mm, and 0.38 vs. 0.43, respectively; p < 0.01; Table 2). Yolk index was among the few egg quality parameters that improved in response to BG supplementation during HS exposure. A significant treatment × period interaction was detected for yolk index (p = 0.05), whereby hens supplemented with 3 g/kg BG (BG3) exhibited a higher yolk index under HS conditions than hens receiving the other dietary treatments, representing an approximately 10% improvement (Table 2). No significant treatment effects or treatment × period interactions were observed for yolk color, breaking strength, or yolk diameter (p > 0.05; Table 2).

**Table 2:** Effects of β-glucan supplementation on the egg quality of laying hens.

Parameter	TN^1^ Ctrl³	TN^1^ BG1⁴	TN^1^ BG2⁵	TN^1^ BG3⁶	HS^2^ Ctrl³	HS^2^ BG1⁴	HS^2^ BG2⁵	HS^2^ BG3⁶	SEM^7^	Trt^8^	Period	Trt × Period
Albumen height, mm	8.5	8.4	8.5	8.9	5.6	5.3	5.8	5.6	0.32	0.64	<0.01	0.80
Yolk color	4.0	3.4	3.4	3.5	3.5	3.7	3.8	3.7	0.29	0.89	0.71	0.53
Haugh unit	92.9	91.9	92.9	95.4	75.8	73.4	77.4	74.8	1.9	0.52	<0.01	0.60
Breaking strength, kg/cm³	4.8	5.5	4.6	5.4	5.0	5.1	4.4	4.8	0.31	0.05	0.22	0.55
Eggshell thickness, mm	0.37	0.37	0.35	0.36	0.35	0.35	0.34	0.34	0.01	0.15	<0.01	0.95
Yolk height, mm	17.4	17.1	17.1	17.4	15.2	15.6	15.9	16.3	0.27	0.16	<0.01	0.15
Yolk diameter, mm	39.0	39.7	43.1	39.4	41.5	41.3	41.5	40.7	1.1	0.17	0.23	0.30
Yolk index	0.45ᵃ	0.43ᵃ	0.40ᵇ	0.44ᵃ	0.36ᶜ	0.38ᶜ	0.38ᶜ	0.41ᵇ	0.01	0.11	<0.01	0.05

1. Period 1, during which laying hens were exposed to TN conditions for 7 days. 2. Period 2, during which laying hens were exposed to cyclic HS conditions for 4 consecutive days. 3. Control hens receiving the basal diet without BG supplementation. 4. Hens received 1 g/kg BG in the diet. 5. Hens received 2 g/kg BG in the diet. 6. Hens received 3 g/kg BG in the diet. 7. Standard error of the mean 8. Treatment.

Means within a row lacking a common superscript differ significantly (p < 0.05).

### Blood gas parameters

The effects of dietary BG supplementation and environmental conditions on blood gas parameters are presented in Table 3. A significant main effect of period was observed, whereby HS increased blood pH, bicarbonate (HCO3), sodium concentration, and oxygen saturation compared with TN conditions (7.52 vs. 7.34, 31.2 vs. 25.0 mmol/L, 151.6 vs. 148.8 mmol/L, and 60.7% vs. 37.7%, respectively; p < 0.05; Table 3). In contrast, HS reduced blood pCO2, potassium concentration, and ionized calcium concentration compared with TN conditions (39.2 vs. 58.8 mmHg, 5.5 vs. 6.6 mmol/L, and 1.44 vs. 1.56 mmol/L, respectively; p < 0.05; Table 3). No significant treatment × period interactions were observed for any blood gas parameter (p > 0.05; Table 3).

**Table 3:** Effects of feeding different levels of β-glucan on blood gas parameters of laying hens.

Parameter	TN^1^ Ctrl³	TN^1^ BG1⁴	TN^1^ BG2⁵	TN^1^ BG3⁶	HS^2^ Ctrl³	HS^2^ BG1⁴	HS^2^ BG2⁵	HS^2^ BG3⁶	SEM^7^	Trt^8^	Period	Trt × Period
pH	7.36	7.30	7.35	7.37	7.52	7.54	7.48	7.55	0.04	0.16	<0.01	0.35
pCO_2_, mmHg	53.2	54.7	53.1	74.5	35.4	37.5	40.7	43.1	5.3	0.02	0.02	0.19
pO_2_, mmHg	25.3	26.2	26.5	25.8	31.7	29.7	30.3	32.5	4.2	0.83	0.16	0.46
HCO_3_, mmol/L	25.5	24.7	24.2	25.7	29.9	31.1	32.6	31.2	1.7	0.45	0.03	0.53
Total CO_2_, mmol/L	29.5	32.8	29.5	32.1	28.6	31.1	33.3	30.4	2.0	0.33	0.33	0.60
Hemoglobin, g/dL	8.5	8.3	8.1	7.8	8.2	8.9	7.8	8.1	0.2	0.63	0.07	0.39
Hematocrit, %	24.2	24.9	24.5	23.2	23.5	25.4	22.2	23.1	0.83	0.36	0.06	0.44
Sodium, mmol/L	149.3	148.5	149.1	148.3	151.2	152.0	151.1	152.3	2.0	0.76	<0.01	0.87
Potassium, mmol/L	6.2	6.9	6.5	6.9	5.5	5.3	5.9	5.2	0.3	0.77	<0.01	0.32
Ionized calcium, mmol/L	1.53	1.59	1.49	1.63	1.45	1.39	1.48	1.44	0.08	0.35	0.02	0.54
Oxygen saturation, %	30.2	40.1	35.6	45.0	70.8	50.5	55.9	65.7	9.2	0.66	0.03	0.35
Chloride, mmol/L	113.2	114.7	112.2	113.1	112.9	113.3	114.5	112.0	1.1	0.43	0.63	0.48

1. Period 1, during which laying hens were exposed to TN conditions for 7 days. 2. Period 2, during which laying hens were exposed to cyclic HS conditions for 4 consecutive days. 3. Control hens receiving the basal diet without BG supplementation. 4.Hens receiving 1 g/kg BG in the diet. 5. Hens receiving 2 g/kg BG in the diet. 6. Hens receiving 3 g/kg BG in the diet. 7. Standard error of the mean 8. Treatment.

Means within a row lacking a common superscript differ significantly (p < 0.05).

### Plasma biochemical parameters

The effects of dietary BG supplementation and environmental conditions on plasma biochemical parameters are presented in Table 4. A significant main effect of period was observed, whereby hens exposed to HS exhibited increased circulating glucose, lactate, alanine transaminase, and LPS concentrations compared with hens maintained under TN conditions (282 vs. 240 mg/dL, 9.5 vs. 5.0 mmol/L, 0.63 vs. 0.40 U/L, and 0.79 vs. 0.68 ng/mL, respectively; p < 0.01; Table 4). In contrast, blood urea nitrogen, haptoglobin, alkaline phosphatase, insulin, serum amyloid A, and glutathione peroxidase concentrations were not significantly affected by environmental conditions (p > 0.05; Table 4).

**Table 4:** Effects of feeding different levels of β-glucan on plasma biochemical parameters of laying hens.

Parameter	TN^1^ Ctrl³	TN^1^ BG1⁴	TN^1^ BG2⁵	TN^1^ BG3⁶	HS^2^ Ctrl³	HS^2^ BG1⁴	HS^2^ BG2⁵	HS^2^ BG3⁶	SEM^7^	Trt^8^	Period	Trt × Period
Glucose, mg/dL	235	242	233	249	287	277	296	270	15.7	0.32	<0.01	0.88
Lactate, mmol/L	5.5	4.9	5.1	4.5	8.9	8.4	11.1	9.5	1.7	0.25	<0.01	0.43
Blood urea nitrogen, mmol/L	1.88	1.69	1.91	1.71	1.58	1.76	1.53	1.68	0.64	0.44	0.76	0.27
Haptoglobin, ng/mL	0.75	0.89	0.94	0.83	1.65	1.70	0.95	1.30	0.73	0.28	<0.01	0.73
Alanine transaminase, U/L	0.39	0.41	0.45	0.35	0.69	0.63	0.65	0.55	0.23	0.65	<0.01	0.46
Alkaline phosphatase, ng/mL	0.29	0.30	0.28	0.33	0.35	0.25	0.29	0.32	0.15	0.89	0.43	0.71
Lipopolysaccharide, ng/mL	0.63	0.70	0.82	0.56	0.83	0.76	0.90	0.66	0.45	0.67	<0.01	0.34
Insulin, ng/mL	7.96	8.76	4.65	5.52	4.50	7.24	6.54	8.63	1.7	0.47	0.31	0.18
Serum amyloid A, ng/mL	265	289	273	256	233	213	251	294	36	0.52	0.35	0.28
Glutathione peroxidase, ng/mL	6.42	4.97	4.55	5.64	5.96	6.13	5.59	5.13	0.46	0.11	0.17	0.21

1. Period 1, during which laying hens were exposed to TN conditions for 7 days. 2. Period 2, during which laying hens were exposed to cyclic HS conditions for 4 consecutive days. 3. Control hens receiving the basal diet without BG supplementation. 4. Hens receiving 1 g/kg BG in the diet. 5. Hens receiving 2 g/kg BG in the diet. 6. Hens receiving 3 g/kg BG in the diet. 7. Standard error of the mean 8.Treatment.

Means within a row lacking a common superscript differ significantly (p < 0.05).

No significant treatment × period interactions or treatment effects were observed for any plasma biochemical parameter (p > 0.05; Table 4), indicating that dietary BG supplementation did not significantly alter these variables under either TN or HS conditions.

### Intestinal morphology

**Figure 4:**
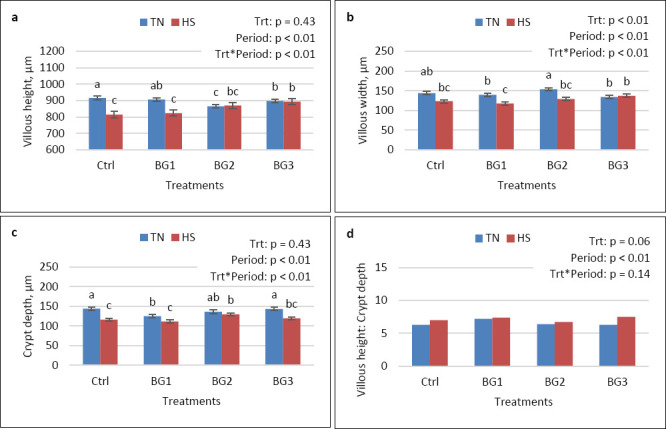
Effects of β-glucan (BG) supplementation on jejunal morphology of laying hens maintained under thermo-neutral (TN) or cyclic heat stress (HS) conditions: (a) villus height, (b) villus width, (c) crypt depth, and (d) villus height-to-crypt depth ratio. Ctrl = control hens receiving the basal diet, BG1 = hens receiving 1 g/kg BG, BG2 = hens receiving 2 g/kg BG, and BG3 = hens receiving 3 g/kg BG. Data are presented as LSM ± SEM.

**Figure 5:**
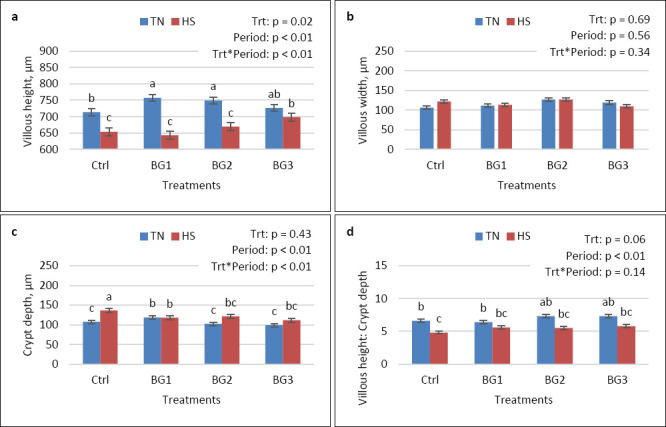
Effects of β-glucan (BG) supplementation on ileal morphology of laying hens maintained under thermo-neutral (TN) or cyclic heat stress (HS) conditions: (a) villus height, (b) villus width, (c) crypt depth, and (d) villus height-to-crypt depth ratio. Ctrl = control hens receiving the basal diet, BG1 = hens receiving 1 g/kg BG, BG2 = hens receiving 2 g/kg BG, and BG3 = hens receiving 3 g/kg BG. Data are presented as LSM ± SEM.

HS adversely affected intestinal morphology, as evidenced by reductions in jejunal villus height, villus width, and crypt depth compared with TN conditions (850 vs. 896 μm, 127 vs. 143 μm, and 119 vs. 137 μm, respectively; p < 0.01; Figures 4a–c). Similarly, HS reduced ileal villus height and the villus height-to-crypt depth ratio compared with TN conditions (666 vs. 737 μm and 5.4 vs. 6.9, respectively; p < 0.01; Figure 5a and d).

Dietary supplementation with 3 g/kg BG (BG3) improved intestinal morphology under HS conditions. Hens receiving BG3 exhibited greater villus height in both the jejunum and ileum than control hens exposed to HS, representing increases of approximately 10% and 7%, respectively (p < 0.01; Figure 4a and Figure 5a).

## DISCUSSION

### Principal findings

The present study demonstrated that dietary BG supplementation partially alleviated several adverse effects of cyclic HS in commercial Hy-Line laying hens. HS significantly increased rectal temperature and negatively affected productive performance, egg quality, blood gas homeostasis, plasma biochemical parameters, and intestinal morphology. Dietary supplementation with BG, particularly at 3 g/kg, improved laying rate, egg mass, FCR, yolk index, and intestinal villus architecture under HS conditions. Notably, intestinal morphology was preserved despite limited effects on blood biochemical indicators, suggesting that maintenance of gut integrity may be a primary mechanism by which BG exerts its beneficial effects during thermal stress.

The preservation of jejunal and ileal villus height by supplementation with 3 g/kg BG during HS represents a novel demonstration of structural intestinal protection in heat-stressed laying hens. These improvements extend beyond the productive performance responses observed in previous studies on laying hens and suggest that maintaining intestinal architecture may be one of the primary mechanisms by which BG contributes to improved resilience during thermal stress.

### BG supplementation and productive performance under HS

To our knowledge, this is among the first studies to demonstrate that short-term dietary supplementation with 3 g/kg BG during the laying phase can partially mitigate the negative effects of cyclic HS on egg production and intestinal villus architecture in commercial Hy-Line hens. These effects were achieved using BG inclusion levels substantially higher than those evaluated in previous laying hen studies. HS is a major constraint to livestock productivity because it compromises animal performance, health, and the expression of genetic potential, even under optimal management conditions. Rising global temperatures are expected to intensify these challenges, further increasing economic losses for livestock producers. Consequently, nutritional interventions have gained considerable attention as practical tools to alleviate the adverse effects of HS. Among these interventions, dietary BG supplementation has been recognized because of its prebiotic properties, ability to support intestinal integrity [10, 11], capacity to modulate beneficial microbiota [14], and potential to attenuate inflammatory responses [13, 15].

Previous studies have demonstrated the beneficial effects of BG supplementation at relatively low dietary inclusion levels during earlier production stages [16]. The present study extends those findings by providing evidence that higher dietary inclusion levels may be required to preserve intestinal integrity and productive performance during acute thermal challenges in the laying phase.

Our results demonstrated that HS induced a marked increase in rectal temperature and significantly impaired productive performance, as evidenced by reductions in feed intake, laying rate, and egg mass, together with a deterioration in FCR. These findings are consistent with the well-documented effects of HS on poultry productivity, whereby elevated ambient temperatures suppress appetite and reduce laying persistency [17, 18]. Egg production declined from 86% under TN conditions to 63% under HS conditions, whereas FCR increased from 1.91 to 2.38. These findings agree with previous reports describing substantial production losses in laying hens exposed to thermal stress [17].

Interestingly, hens receiving BG supplementation during HS exhibited higher hen-day egg production, greater egg mass, and improved FCR than unsupplemented hens. These findings suggest that BG supplementation enabled laying hens to maintain productive performance closer to normal physiological levels despite thermal challenge. Similar observations have been reported by Ezzat *et al*. [16], who demonstrated that BG supplementation improved FCR and egg production in laying hens exposed to chronic HS. Likewise, our previous study in broiler chickens exposed to acute HS demonstrated that supplementation with 3 g/kg BG increased feed intake by approximately 23% and improved body weight gain relative to control birds [10]. Similar improvements in growth performance, feed efficiency, and body weight gain in heat-stressed broilers supplemented with BG have also been reported [19].

The beneficial effects of BG on productive performance are likely multifactorial. Improved intestinal health and nutrient-absorption capacity [20, 21] may support egg formation and nutrient utilization under HS conditions. Furthermore, the anti-inflammatory effects of BG reported previously [22, 23] may help maintain feed intake and metabolic efficiency during thermal stress.

### BG supplementation and egg quality under HS

In the present study, HS adversely affected both external and internal egg quality characteristics. Significant reductions were observed in albumen height, Haugh unit, eggshell thickness, yolk height, and yolk index under HS conditions. These findings are consistent with previous studies reporting that HS disrupts acid-base balance and calcium metabolism, leading to thinner eggshells and poorer albumen quality in laying hens [17, 24]. Other studies have similarly documented reductions in egg quality in hens exposed to HS [25, 26], with decreases in eggshell thickness closely associated with reduced ionized calcium concentrations [24].

Notably, hens supplemented with 3 g/kg BG under HS conditions exhibited an approximately 10% increase in yolk index compared with control hens, indicating improved yolk structural integrity. However, BG supplementation did not significantly influence eggshell thickness, shell breaking strength, or albumen height during HS exposure. It is possible that the severity of thermal stress outweighed the potential benefits of BG on egg formation. Maintaining egg quality during severe heat exposure remains challenging because hens must divert substantial energy toward thermoregulation, at the expense of nutrient allocation to egg production.

In contrast, studies conducted under TN conditions have reported more pronounced benefits of yeast- and BG-based supplements on egg quality characteristics. For example, Park *et al*. [27] reported significant linear improvements in egg weight, albumen height, Haugh unit, and eggshell thickness in laying hens receiving increasing levels of brewer’s yeast hydrolysate, a product rich in BG.

The effects of BG supplementation on productive performance and egg quality are not entirely consistent across studies. For example, Hashim *et al*. [28] reported improvements in eggshell characteristics, including shell thickness and shell weight, whereas other studies observed limited or no effects on egg production or egg quality parameters [29]. Such variability may be attributed to differences in BG source, dietary inclusion level, environmental conditions, bird age, and supplementation duration.

### Blood gas responses and calcium-related changes under HS

HS-induced respiratory alkalosis may directly contribute to impaired eggshell formation. Increased panting during HS promotes excessive loss of carbon dioxide, resulting in elevated blood pH and disruption of calcium homeostasis. Consequently, the availability of ionized calcium, the biologically active form required for eggshell mineralization, is reduced. Lower ionized calcium concentrations may limit calcium deposition within the shell gland [24], thereby contributing to the reduction in eggshell thickness observed during HS.

The current findings reflect the hyperventilation response used by birds to dissipate excess body heat. This physiological adaptation causes substantial carbon dioxide loss and an increase in blood pH. Our results are consistent with previous reports showing that HS induces respiratory alkalosis in poultry [24, 30, 31].

### Plasma biochemical responses to HS and BG supplementation

HS triggered significant alterations in metabolic indicators. Circulating glucose and lactate concentrations increased by approximately 18% and 90%, respectively, in HS hens compared with TN hens. It has been proposed that skeletal muscle contributes to metabolic adaptation during stress by releasing lactate, which can subsequently be utilized as an energy source by non-immune tissues. This mechanism may preserve glucose availability for immune cells and reflects a metabolic shift similar to the Warburg effect [10, 32, 33].

During immune activation induced by HS, muscle catabolism may also serve as a compensatory mechanism for supplying gluconeogenic substrates and essential amino acids required for acute-phase protein synthesis [34, 35], while simultaneously contributing to Adenosine triphosphate generation through enhanced glycolytic activity [36].

Circulating alanine transaminase concentrations were also elevated in HS hens compared with TN hens. Similar findings have previously been reported in broiler chickens exposed to HS [10, 37]. Despite improvements in productivity and intestinal morphology, BG supplementation did not significantly affect the blood biomarkers measured in the present study.

### Intestinal morphology and gut barrier protection

HS imposes a substantial physiological burden on animals by redistributing blood flow away from the gastrointestinal tract toward peripheral tissues to facilitate heat dissipation [38, 39]. Reduced intestinal perfusion and oxygen supply compromise epithelial integrity and disrupt tight junction proteins, thereby increasing intestinal permeability, commonly referred to as “leaky gut” [18, 39, 40]. As a consequence, luminal endotoxins, particularly LPS derived from Gram-negative bacteria, may translocate into systemic circulation [41, 42], triggering inflammatory responses.

Our results demonstrated that HS markedly impaired intestinal morphology. Control hens exposed to HS exhibited shorter and narrower villi together with reduced crypt depth in both the jejunum and ileum compared with TN hens, indicating intestinal mucosal atrophy. Elevated body temperature and altered blood flow distribution are known to damage intestinal epithelial tissues, resulting in reductions in villus height and absorptive surface area [10, 43].

Importantly, supplementation with 3 g/kg BG alleviated several of the adverse effects of HS. Hens receiving BG3 exhibited greater villus height in both the jejunum and ileum than unsupplemented hens exposed to HS. The preservation of jejunal and ileal villus height by supplementation with 3 g/kg BG under HS conditions represents a novel demonstration of structural intestinal protection in heat-stressed laying hens and extends beyond the productive performance responses reported previously in laying hen studies.

### Mechanistic interpretation of BG-mediated HS mitigation

The improvement in intestinal morphology suggests that BG helps preserve intestinal integrity during thermal challenge. Unlike many nutritional additives that require prolonged supplementation or exhibit benefits primarily under TN conditions, supplementation with 3 g/kg BG provided measurable intestinal protection following a 28-day pre-feeding period and only 4 days of HS exposure. This finding highlights the potential utility of BG as a short-term nutritional strategy during heat-wave events.

Our previous work demonstrated that broilers supplemented with 3 g/kg BG under acute HS exhibited marked improvements in intestinal morphometric parameters, including increased villus height and width within the small intestine [10]. Similarly, Park *et al*. [27] reported that brewer’s yeast hydrolysate supplementation promoted beneficial shifts in the intestinal microbiota of laying hens, characterized by increased populations of *Lactobacillus* spp. and reduced counts of *E. coli*, changes generally associated with improved intestinal health and morphology. Consistently, Zhang *et al*. [19] reported reduced ileal *E. coli* colonization in HS broilers receiving BG supplementation, suggesting a more stable intestinal environment.

Although direct measurements of intestinal permeability, inflammatory cytokines, and barrier-function markers were not performed, the combination of improved villus morphology and numerically lower circulating LPS concentrations suggests that BG may contribute to the maintenance of intestinal barrier-function during HS. In addition, the positive effects of BG on intestinal integrity may be partially mediated through enhanced monocyte and macrophage activity, which can stimulate glucagon-like peptide-2 secretion [44]. Glucagon-like peptide-2 promotes intestinal stem cell proliferation and reduces intestinal permeability to pathogens [45, 46]. By reinforcing barrier integrity and supporting a favorable intestinal microbial community, BG likely reduces HS-associated intestinal damage. Under HS conditions, preservation of gut barrier-function may limit endotoxin translocation and excessive immune activation, thereby preventing further tissue injury.

### Practical implications for commercial laying hens

The present study makes several important contributions to understanding nutritional strategies to mitigate HS in laying hens. First, it demonstrates a dose-dependent response to dietary BG supplementation under both TN and HS conditions during the laying phase. Second, it provides direct quantitative evidence of improved intestinal morphology, indicating partial preservation of intestinal structural integrity during thermal challenge. Third, BG supplementation under HS was associated with selective improvements in productive performance, including egg production, feed efficiency, and yolk index, without inducing widespread alterations in blood biochemical parameters.

From a practical standpoint, supplementation with 3 g/kg BG may be a promising nutritional strategy to support productive performance and intestinal integrity in laying hens exposed to HS. Unlike many feed additives evaluated under controlled conditions, BG provided measurable benefits during a relatively short-term HS challenge, suggesting potential utility during seasonal heat waves. Nevertheless, adoption in commercial production systems should consider cost-effectiveness and return on investment. Furthermore, the scalability of this approach requires evaluation under commercial field conditions, particularly in large-scale production systems where birds are simultaneously exposed to multiple environmental and management-related stressors.

### Strengths of the study

The present study has several strengths. It evaluated multiple dietary inclusion levels of BG during the laying phase under both TN and HS conditions, enabling assessment of dose-dependent responses. In addition, the study integrated productive performance, egg quality, blood physiological responses, plasma biochemical indicators, and quantitative intestinal morphometry within a single experimental model. The direct demonstration of improved intestinal villus architecture under HS conditions provides mechanistic support for the observed improvements in productive performance and represents one of the major novel contributions of this work.

### Study limitations and future directions

Several limitations should be acknowledged. First, the HS protocol represented an acute thermal challenge (4 h/day for 4 days) rather than a chronic HS model. Second, reductions in feed intake during HS may have confounded some treatment responses. Third, although intestinal morphology improved with BG supplementation, measurements of intestinal microbiota composition, barrier-function markers, inflammatory cytokines, short-chain fatty acids, and oxidative stress indices were not performed. Finally, nutrient digestibility was not evaluated, limiting the interpretation of the functional mechanisms underlying the observed improvements in productive performance and intestinal morphology.

Future studies should investigate the effects of BG supplementation during prolonged HS exposure and evaluate its interactions with intestinal microbiota, immune responses, oxidative stress pathways, nutrient digestibility, and gut barrier-function. Such studies would provide a more comprehensive understanding of the biological mechanisms underlying the beneficial effects of BG under thermal stress and facilitate the development of optimized nutritional strategies for commercial poultry production.

## CONCLUSION

Cyclic HS markedly impaired the productive performance, egg quality, physiological homeostasis, and intestinal morphology of commercial Hy-Line laying hens. Exposure to HS increased rectal temperature and altered blood gas balance, while significantly reducing feed intake, laying rate, egg mass, albumen height, Haugh unit, eggshell thickness, yolk height, and yolk index. HS also induced metabolic disturbances, including increases in circulating glucose, lactate, alanine aminotransferase, and LPS concentrations, and caused substantial deterioration in intestinal morphology, as evidenced by reduced jejunal and ileal villus dimensions.

Dietary supplementation with BG partially mitigated several of these adverse effects. In particular, supplementation with 3 g/kg BG improved laying rate, egg mass, and FCR under HS conditions and enhanced yolk index by approximately 10% compared with unsupplemented hens. Moreover, BG supplementation preserved intestinal morphology during thermal challenge, resulting in greater jejunal and ileal villus height under HS conditions. The maintenance of intestinal architecture occurred despite minimal effects on most blood biochemical parameters, suggesting that preservation of gut integrity may represent a primary mechanism through which BG supports productive performance during heat exposure.

The findings of this study demonstrate that dietary BG supplementation at 3 g/kg can serve as a practical nutritional strategy to improve the resilience of laying hens exposed to acute cyclic HS. The observed improvements in productivity and intestinal morphology highlight the importance of supporting gut health to reduce the detrimental consequences of thermal stress. Collectively, these results provide new evidence that higher dietary inclusion levels of BG during the laying phase may help maintain productivity and intestinal integrity under challenging environmental conditions. Further research under chronic HS and commercial production settings is warranted to confirm the long-term efficacy and economic feasibility of this nutritional intervention.

## DATA AVAILABILITY

The supplementary data can be made available from the corresponding author upon request.

## AUTHORS’ CONTRIBUTIONS

MA, ZMHM, MAQ, MAM, ARAF, and AA: Conceptualization, methodology, data analysis, data interpretation, manuscript drafting, supervision and critical revision of the manuscript for important intellectual content. All authors have read and approved the final manuscript.
